# The Use of Recommended Communication Techniques by Maryland Family Physicians and Pediatricians

**DOI:** 10.1371/journal.pone.0119855

**Published:** 2015-04-09

**Authors:** Darien J. Weatherspoon, Alice M. Horowitz, Dushanka V. Kleinman, Min Qi Wang

**Affiliations:** 1 University of Illinois at Chicago, College of Dentistry, Department of Pediatric Dentistry, Chicago, Illinois, United States of America; 2 University of Maryland, School of Public Health, College Park, Maryland, United States of America; UNC School of Dentistry, University of North Carolina-Chapel Hill, UNITED STATES

## Abstract

**Background:**

Health literacy experts and the American Medical Association have developed recommended communication techniques for healthcare providers given that effective communication has been shown to greatly improve health outcomes. The purpose of this study was to determine the number and types of communication techniques routinely used by Maryland physicians.

**Methods:**

In 2010, a 30-item survey was mailed to a random sample of 1,472 Maryland family physicians and pediatricians, with 294 surveys being returned and usable. The survey contained questions about provider and practice characteristics, and 17 items related to communication techniques, including seven basic communication techniques. Physicians’ use of recommended communication techniques was analyzed using descriptive statistics, analysis of variance, and ordinary least squares regression.

**Results:**

Family physicians routinely used an average of 6.6 of the 17 total techniques and 3.3 of the seven basic techniques, whereas pediatricians routinely used 6.4 and 3.2 techniques, respectively. The use of simple language was the only technique that nearly all physicians routinely utilized (Family physicians, 91%; Pediatricians, 93%). Physicians who had taken a communications course used significantly more techniques than those who had not. Physicians with a low percentage of patients on Medicaid were significantly less likely to use the recommended communication techniques compared to those providers who had high proportion of their patient population on Medicaid.

**Conclusions:**

Overall, the use of recommended communication techniques was low. Additionally, many physicians were unsure of the effectiveness of several of the recommended techniques, which could suggest that physicians are unaware of valuable skills that could enhance their communication. The findings of this study suggest that communications training should be given a higher priority in the medical training process in the United States.

## Introduction

Healthy People 2020 (HP 2020) highlights the national importance of effective patient-provider communication [1]. Health Communication and Health Information Technology Objective 2 of HP 2020 aims to “increase the proportion of persons who report that their health care providers have satisfactory communication skills [1].” Strong communication skills are an essential component of patient-centered care, an approach to care that actively involves patients in the treatment decision-making process [2]. Patient-centered care has received increased attention in the past decade in the United States (U.S.), and will likely become an even more important model of patient care under the Patient Protection and Affordable Care Act of 2010 [2].

Communication is also an important part of health literacy, which is defined as “the degree to which individuals have the capacity to obtain, process, and understand basic health information and services needed to make appropriate health decisions [3].” The first assessment of adult health literacy in the U.S. found that a majority of adults lack the necessary health literacy skills to fully understand or make decisions based on health information that they receive [4]. Low health literacy can negatively impact an individual’s health by causing non-compliance to medication; poor adherence to recommended treatment; delayed decision making; and poor management of chronic diseases, including oral health conditions [5–9].

Given that low health literacy is nearly ubiquitous and that low health literacy has been linked to poorer health outcomes, effective physician communication becomes especially critical to ensure that patients fully understand the health information presented to them and to increase the chances for positive health outcomes [1,10,11]. Studies examining physicians’ communication skills have shown that effective communication is related to greater patient adherence to treatment and ultimately improved patient health outcomes including improved management of chronic diseases, such as diabetes and hypertension [12–14].

Despite the benefits of effective communication, this skillset is often underutilized by physicians [11,15,16]. The overestimation of patients’ health literacy by physicians is one likely cause of less-than-optimal communication with patients [10]. In other words, physicians may not always place as much focus on communicating effectively with patients because they may incorrectly assume that patients medical knowledge and comprehension is greater than it actually is [10]. An additional barrier to effective provider communication could be physicians’ unawareness of the negative impact that poor health literacy has on patient self-management and health outcomes [17].

In an effort to improve provider communication, health literacy experts and the American Medical Association (AMA) have recommended 17 communication techniques [11]. Some of these techniques include using simple language; handing out printed materials; speaking more slowly; presenting 2–3 concepts at a time; asking patients how they will follow instructions at home; and asking patients to repeat information (teach back) [11]. A 2007 study on health care providers’ use of communication techniques found that while some techniques, such as using simple language (94.7%) were commonly used; the majority of techniques recommended by health literacy experts were not routinely used by providers [11]. To further complicate this issue, medical students receive very little practical training in communication, and during residency training when most patient contact occurs, training in communication receives very low priority [2].

Understanding the use of communication techniques by family physicians and pediatricians is especially critical because these practitioners see a wide range of patients, making them a valuable resource in promoting other aspects of health, such as oral health [18]. Therefore, the overall aims of this study are to determine: 1) the number and types of communication techniques Maryland pediatricians and family practice physicians use on a routine basis 2) their perception of the effectiveness of the recommended communication techniques and 3) factors associated with their use of these techniques. This survey of Maryland physicians was part of a comprehensive, state-wide assessment of oral health literacy and communication techniques that was conducted. The proposed study aims were achieved, and the results will serve as a baseline for future studies aimed at further assessing and improving provider communication in the state of Maryland.

## Materials and Methods

### Sampling Strategy

This cross-sectional study used a 30-item, self-administered questionnaire on dental caries prevention and communication techniques that was mailed to a random sample of 1,472 family practice physicians (family physicians) and pediatricians in the state of Maryland in June and July of 2010. This study will focus on the responses related to the use of communication techniques. The questionnaire contained 17 items concerning the routine use of recommended communication techniques, which were adopted from a questionnaire used by Rozier et al. and the recommended techniques from the health literacy experts and the AMA [11,19]. It was used to assess the perceived effectiveness, routine use, and characteristics associated with of the use of recommended communication techniques by Maryland physicians. This questionnaire has been used in previous studies to assess the use of recommended communication techniques by Maryland dentists and dental hygienists [20,21].

Prior to mailing the survey, the instrument was first pilot-tested with family physicians and pediatricians. After receiving feedback from the physicians, necessary revisions were made and the survey was printed in a format that allowed it to be pre-paid and returned without an envelope.

The 17 communication questions are grouped into the following five domains: Interpersonal communications, Teach-back method, Patient-friendly materials and aids, Assistance, and Patient-friendly practice. The seven basic communication techniques are found in the first two domains (Interpersonal communications and Teach-back method). Physicians were asked how often they used each of the 17 communication techniques in a typical workweek using a Likert-type scale with the following five response options: always, most of the time, occasionally, rarely, and never. The responses were scored from 5 = “always” to 1 = “never”. Additionally, for each technique the physicians were asked whether they thought that the technique was effective using the following response options:” yes”, “no”, or “do not know.”

The outcome variable for the analysis of the communication techniques was a count of the routine use of the 17 communication techniques. Additionally, seven of the 17 recommended communication techniques were used as a separate variable (the seven basic techniques). For the purpose of analysis, “routine use” was defined as responses of “always” or “most of the time” (verses the responses of “never,” “rarely,” or “occasionally”). Respondents were also asked additional questions regarding provider characteristics (i.e.: if they’ve taken a communication course) and practice characteristics (i.e.: if they’ve assessed their office for user-friendliness).

The study sample was obtained using the membership lists of Maryland Academy of Family Physicians (MAFP) and the Maryland American Academy of Pediatrics (MDAAP). An initial mailing consisted of the full survey questionnaire along with a cover letter explaining the survey that was signed by either the President of the MAFP or MDAAP. Three weeks after the first mailing, a second complete mailing was sent to non-respondents. Approximately three weeks after the second mailing, a postcard was mailed to remaining non-respondents. The executive director of each organization was asked to send a blast email reminder to urge all members to respond to the survey.

### Ethics Statement

The Institutional Review Board, University of Maryland, College Park, reviewed and approved this study. The physicians were informed that their participation in this study was completely voluntary, and passive informed consent was obtained by participants completing and returning the survey.

### Data Analysis

Data was analyzed using SAS Version 9.3 (SAS Institute, Inc., Cary, NC.). Separate analyses were conducted for family physicians and pediatricians because they were from different sampling frames. The statistical analyses included distributions (frequencies and percentages) of physician characteristics, routine use of techniques, and perceived effectiveness of techniques. Additionally, the associations between all demographic variables and the mean number of communication techniques used were examined using the Analysis of Variance (ANOVA). For the ANOVA, the selected predictor variables were used as the independent variable and the mean number of communication techniques routinely used was the dependent variable. Finally, ordinary least squares regression was used to analyze the association between selected predictor variables (i.e.: provider and practice characteristics) as the independent variables, and the count of communication techniques routinely used as the dependent variable. The level of significance was set at p<0.05.

## Results

### Sample results and characteristics

Of the 1,472 surveys mailed to Maryland family physicians and pediatricians, 415 were returned and of the total surveys returned, 294 were usable (consisting of 215 pediatricians and 79 family physicians) yielding an effective response rate of 20%. Table 1 displays the characteristics of the sample in total, and by family physician and pediatrician providers. The majority of respondents were white (75%), female (62%), private practitioners (67%), and practiced in a group setting (58%). These characteristics were similar for both family physicians and pediatricians. Thirty percent of pediatricians graduated prior to 1980 compared to only 12% of family physicians. Nearly half of all respondents had assessed their office to determine if it was user friendly. Additionally, about half of both family physicians and pediatricians reported to have previously taken a communications course, and a similar proportion of respondents were interested in attending a communications continuing education course.

**Table 1:** Distribution of physicians’ characteristics in total and by family physician and pediatrician providers.

Characteristics	Family Physicians, n = 79		Pediatricians, n = 215		Total, N = 294	
	n ^a^	Percentage (%)	n ^a^	Percentage (%)	N ^a^	Percentage (%)
**Year of Graduation**						
1979 or Prior	9	12.7	59	30.0	68	25.4
1980–1989	27	38.0	56	28.4	83	31.0
1990–1999	18	25.4	45	22.8	63	23.5
2000 or Later	17	23.9	37	18.8	54	20.2
**Practice Setting**						
Solo Practice	12	15.4	23	10.9	35	12.1
Group Practice	41	52.6	128	60.7	169	58.5
All Other	25	32.1	60	28.4	85	29.4
**Occupation**						
Private Practice	49	63.6	145	68.7	194	67.4
All Other	28	36.4	66	31.3	94	32.6
**Race/Ethnicity**						
White	57	74.0	158	76.0	215	75.7
Black	5	6.6	19	9.1	24	8.5
All Other	14	18.4	31	14.9	45	15.9
**Gender**						
Female	48	62.3	132	62.0	180	62.1
Male	29	37.7	81	38.0	110	37.9
**Type of insurance for child patients**						
Medicaid/SCHIP	71	28.8*	200	35.2*	271	33.5*
Private Insurance	71	63.9*	202	60.5*	273	61.4*
Out of Pocket	66	7.3*	190	4.5*	256	5.2*
**Ever taken a communications course**						
Yes	41	53.2	103	48.4	144	49.7
No	36	46.8	110	51.6	146	50.3
**Interested in attending communications continuing education course**						
Yes	30	39.0	93	44.1	123	42.7
No	47	61.0	118	55.9	165	57.3
**Ever assessed office to determine if user-friendly**						
Yes	33	47.1	101	49.5	134	48.9
No	37	52.9	103	50.5	140	51.1

^a^ sample size for each variable may not add up to overall sample size due to missing values

*Mean percentage

### Descriptive results for communication techniques used

Tables 2 and 3 display the percentage distribution for each of the five possible Likert-scale responses and the mean response score for each of the 17 communications techniques grouped into five domains for family physicians and pediatricians, respectively. The first seven techniques are grouped into the “Interpersonal communication” and “Teach-back methods” domains, and are considered to be the basic communications techniques that every health provider should routinely use. Family physicians reported routinely using on average 6.6 of the 17 total techniques and 3.3 of the seven basic techniques, whereas pediatricians reported routinely using 6.4 and 3.2 techniques, respectively (data not shown). The frequency of use varied considerably across the 17 techniques and five domains. The use of simple language was the only basic technique that was routinely used (used “always” or “most of the time”) by nearly all of the respondents (Family physicians, 91%; Pediatricians, 93%) and the technique with the highest mean Likert-scale score (Family physicians, 4.27; Pediatricians, 4.23). Limiting the number of concepts presented, speaking slowly, and writing or printing out instructions were other techniques routinely used by at least 60% of family physicians and pediatricians.

**Table 2:** Percentage distribution of techniques used routinely by family physicians and mean Likert scale scores. *

Communication Technique, Domain and Item	Sample Size, n	Percent Distribution (%)					Mean ^a^ Score
		Always (5)	Most of the Time (4)	Occasionally (3)	Rarely (2)	Never (1)	
**Interpersonal Communication** ^**b**^							
Limit the number of concepts presented at a time (2 to 3)	67	7.46	65.67	23.88	1.49	1.49	3.76
Ask patients whether they would like a family member or friend accompany them in the discussion	68	0.0	11.76	51.47	26.47	10.29	2.65
Draw pictures or use printed illustrations	69	4.35	27.54	47.83	14.49	5.80	3.10
Speak slowly	67	10.45	55.22	28.36	4.48	1.49	3.69
Use simple language	67	38.81	52.24	7.46	0.0	1.49	4.27
**Teach-back Method** ^**b**^							
Ask patients to repeat back information or instructions	68	2.94	29.41	48.53	13.24	5.88	3.10
Ask patients to tell you what they will do at home to follow instructions	68	1.47	29.41	48.53	10.29	10.29	3.01
**Patient-friendly materials and aids**							
Use video or DVD	68	0.0	0.0	8.82	23.53	67.65	1.41
Hand out printed materials	68	22.06	33.82	32.35	8.82	2.94	3.63
Use models or x-rays to explain	69	2.90	7.25	43.48	27.54	18.84	2.48
**Assistance**							
Underline key points on print material	68	10.29	20.59	38.24	17.65	13.24	2.97
Follow-up with patients by telephone to check understanding and adherence	68	0.0	8.82	38.24	35.29	17.65	2.38
Read instructions out loud	68	19.12	38.24	26.47	8.82	7.35	3.53
Ask other office staff to follow up with patients for post-care instructions	68	2.94	14.71	32.35	32.35	17.65	2.53
Write or printout instructions	68	25.00	36.76	35.29	0.0	2.94	3.81
**Patient-friendly practice**							
Refer patients to the Internet or other sources of information	69	2.90	34.78	46.38	10.14	5.80	3.19
Use a translator or interpreter when needed	69	27.54	21.74	23.19	20.29	7.25	3.42

*Some groups of percentages do not add up to 100% due to rounding

^a^ Mean score on a five-point Likert scale ranging from 5 (“always”) to 1 (“never”)

^b^ Items in the “Interpersonal Communication” and Teach-back Method” categories constitute the seven basic communication techniques

**Table 3:** Percentage distribution of techniques used routinely by pediatricians and mean Likert scale scores. *

Communication Technique, Domain and Item	Sample Size, n	Percent Distribution (%)					Mean ^a^ Score
		Always (5)	Most of the Time (4)	Occasionally (3)	Rarely (2)	Never (1)	
**Interpersonal Communication** ^**b**^							
Limit the number of concepts presented at a time (2 to 3)	190	9.47	61.58	21.05	6.32	1.58	3.71
Ask patients whether they would like a family member or friend accompany them in the discussion	194	1.55	6.19	26.29	32.99	32.99	2.10
Draw pictures or use printed illustrations	192	3.65	21.35	47.92	22.40	4.69	2.97
Speak slowly	192	14.06	59.38	21.35	3.13	2.08	3.80
Use simple language	194	32.99	60.31	4.64	1.03	1.03	4.23
**Teach-back Method** ^**b**^							
Ask patients to repeat back information or instructions	192	3.13	24.48	43.23	23.44	5.73	2.96
Ask patients to tell you what they will do at home to follow instructions	192	7.29	20.31	32.81	29.69	9.90	2.85
**Patient-friendly materials and aids**							
Use video or DVD	193	0.0	2.07	5.70	18.13	74.09	1.36
Hand out printed materials	191	14.14	42.93	34.03	6.81	2.09	3.60
Use models or x-rays to explain	193	1.04	8.29	26.42	38.86	25.39	2.21
**Assistance**							
Underline key points on print material	192	4.69	34.90	28.13	19.79	12.50	2.99
Follow-up with patients by telephone to check understanding and adherence	194	2.58	9.28	43.81	26.29	18.04	2.52
Read instructions out loud	193	13.99	38.86	21.24	15.03	10.88	3.30
Ask other office staff to follow up with patients for post-care instructions	192	3.13	11.46	33.85	32.81	18.75	2.47
Write or printout instructions	193	10.88	50.78	31.09	5.70	1.55	3.64
**Patient-friendly practice**							
Refer patients to the Internet or other sources of information	192	2.08	20.83	55.73	17.19	4.17	2.99
Use a translator or interpreter when needed	189	28.57	20.11	21.69	20.63	8.99	3.39

*Some groups of percentages do not add up to 100% due to rounding

^a^ Mean score on a five-point Likert scale ranging from 5 (“always”) to 1 (“never”)

^b^ Items in the “Interpersonal Communication” and Teach-back Method” categories constitute the seven basic communication techniques

Table 4 shows the physicians’ perceived effectiveness of the communications techniques. Using simple language was the technique that both family physicians and pediatricians thought was most effective (family physicians, 81.36%; pediatricians, 85.63%). Limiting the number of concepts, asking patients to repeat back information, writing/printing out instructions, and using a translator/interpreter were techniques that approximately 70% or more family physicians and pediatricians thought were effective. Additionally, about 70% of pediatricians also thought that drawing pictures/using printed illustrations and speaking slowly were effective techniques. Physicians were most unsure about the effectiveness of using videos/DVDs (family physicians, 77.36%; pediatricians, 75%). Over half of family physicians were also unsure about the effectiveness of using models and x-rays to explain health issues and referring patients to the internet or other sources of information, while over half of pediatricians were unsure about the effectiveness of asking their patients if they would like a family member to accompany them and referring patients to the internet or other sources of information.

**Table 4:** Percentage distribution of the perceived effectiveness of techniques used routinely. *

Communication Technique	Family Physicians				Pediatricians			
	n	Yes (%)	No (%)	Unsure (%)	n	Yes (%)	No (%)	Unsure (%)
**Interpersonal Communication** ^**a**^								
Limit the number of concepts presented at a time (2 to 3)	58	74.14	0.0	25.86	160	75.63	1.88	22.50
Ask patients whether they would like a family member or friend	55	58.18	0.0	41.82	141	41.84	5.67	52.48
Draw pictures or use printed illustrations	58	58.62	1.72	39.66	154	71.43	0.65	27.92
Speak slowly	58	60.34	5.17	34.48	164	71.95	1.83	26.22
Use simple language	59	81.36	0.0	18.64	160	85.63	0.63	13.75
**Teach-back Method** ^**a**^								
Ask patients to repeat back information or instructions	56	73.21	3.57	23.21	157	68.79	3.82	27.39
Ask patients to tell you what they will do at home to follow instructions	55	61.82	0.0	38.18	152	59.21	1.97	38.82
**Patient-friendly materials and aids**								
Use video or DVD	53	15.09	7.55	77.36	136	22.79	2.21	75.00
Hand out printed materials	59	57.63	3.39	38.98	160	59.38	6.88	33.75
Use models or x-rays to explain	55	41.82	3.64	54.55	148	52.70	2.03	45.27
**Assistance**								
Underline key points on print material	58	48.28	3.45	48.28	153	53.59	3.92	42.48
Follow-up with patients by telephone to check understanding and adherence	59	59.32	1.69	38.98	150	68.67	2.00	29.33
Read instructions out loud	58	67.24	0.0	32.76	157	52.23	5.73	42.04
Ask other office staff to follow up with patients for post-care instructions	57	49.12	1.75	49.12	149	49.66	4.70	45.64
Write or printout instructions	59	69.49	0.0	30.51	160	77.50	1.25	21.25
**Patient-friendly practice**								
Refer patients to the Internet or other sources of information	58	43.10	5.17	51.72	156	40.38	5.77	53.85
Use a translator or interpreter when needed	57	73.68	0.0	26.32	148	77.03	0.68	22.30

*Some groups of percentages do not add up to 100% due to rounding

^a^ Items in the “Interpersonal Communication” and Teach-back Method” categories constitute the seven basic communication techniques

### Variables associated with routine use of communications techniques

Bivariate analysis of the routine use of communication techniques according to provider and practice characteristics are displayed in Tables 5 and 6. Family physicians that had taken a communications course outside of medical school used significantly more of the 17 recommended communications techniques on average than those who had not (7.71 vs. 5.50; P<0.01). The mean number of 17 and seven basic techniques did not differ significantly by other provider or practice characteristics for family physicians.

**Table 5:** Bivariate analysis of predictor variables and mean communication techniques used routinely by family physicians.

Variable	Sample size (Number, %) ^a^	17 communication techniques (n = 79)		Seven basic communication techniques (n = 79)	
		Mean number of techniques used	Analysis of variance (P- value)	Mean number of techniques used	Analysis of variance (P- value)
**Year of Graduation**			0.43		0.91
1979 or prior	8 (12.9)	5.62		3.25	
1980–1989	22 (35.5)	6.54		3.22	
1990–1999	17 (27.4)	5.64		3.11	
2000-Onward	15 (24.2)	7.46		3.53	
**Race/Ethnicity**			0.10		0.72
White	48 (72.7)	6.14		3.35	
Black	4 (6.1)	6.25		2.75	
All Other	14 (21.2)	8.28		3.50	
Gender			0.32		0.99
Female	43 (64.2)	6.95		3.37	
Male	24 (35.8)	6.12		3.37	
**Ever taken a communications course**			0.002*		0.16
Yes	35 (52.2)	7.71		3.62	
No	32 (47.8)	5.50		3.09	
**Assessed office for user friendliness**			0.43		0.98
Yes	32 (47.8)	6.78		3.21	
No	35 (52.2)	6.14		3.22	
**Percentage of pediatric patients with Medicaid**			0.17		0.13
0 to 25%	37 (60.7)	5.89		3.00	
26 to 50%	9 (14.8)	7.22		3.56	
51% to 75%	5 (8.2)	7.20		4.00	
76% to 100%	10 (16.4)	8.20		4.20	
**Practice setting**			0.67		0.69
Solo practice	12 (17.7)	6.08		3.67	
Group practice	33 (48.5)	6.39		3.30	
All other	23 (33.8)	7.04		3.17	
**Primary occupation**			0.45		0.39
Private practice	41 (61.2)	6.36		3.46	
All other	26 (38.8)	7.00		3.12	

^a^ The sample size for each variable may not be equal to the overall sample size because of missing values

*P <0.01

**Table 6:** Bivariate analysis of predictor variables and mean communication techniques used routinely by pediatricians.

Variable	Sample size (Number, %) ^a^	17 communication techniques (n = 215)		Seven basic communication techniques (n = 215)	
		Mean number of techniques used	Analysis of variance (P- value)	Mean number of techniques used	Analysis of variance (P- value)
**Year of Graduation**			0.04*		0.03*
1979 or prior	53 (29.8)	7.15		3.66	
1980–1989	47 (26.4)	5.91		2.91	
1990–1999	42 (23.6)	5.62		2.93	
2000-Onward	36 (20.2)	6.92		3.42	
**Race/Ethnicity**			0.20		0.44
White	143 (76.1)	6.25		3.14	
Black	17 (9.0)	7.59		3.59	
All Other	28 (14.9)	6.07		3.36	
**Gender**			0.57		0.99
Female	120 (62.2)	6.45		3.22	
Male	73 (37.8)	6.21		3.22	
Ever taken a communications course			0.01**		<.0001*
Yes	90 (46.6)	6.96		3.67	
No	103 (53.4)	5.84		2.83	
**Assessed office for user friendliness**			0.001***		0.001***
Yes	96 (50.0)	7.11		3.60	
No	96 (50.0)	5.69		2.86	
**Percentage of pediatric patients with Medicaid**			0.04*		0.01*
0 to 25%	96 (52.2)	6.03		3.05	
26 to 50%	37 (20.1)	6.41		3.16	
51% to 75%	21 (11.4)	6.57		3.33	
76% to 100%	30 (16.3)	7.80		4.03	
**Practice setting**			0.001***		0.01**
Solo practice	22 (11.5)	5.09		2.77	
Group practice	120 (62.8)	6.10		3.10	
All other	49 (25.7)	7.65		3.76	
**Primary occupation**			0.002**		0.001***
Private practice	137 (71.4)	5.94		3.00	
All other	55 (28.6)	7.42		3.76	

^a^ The sample size for each variable may not be equal to the overall sample size because of missing values

*P <0.05

**P≤0.01

***P≤0.001

For pediatricians, a significant association was observed between year of graduation and the mean number of techniques used. Pediatricians that graduated prior to 1980 or after 1999 reported using significantly more of the 17 and basic techniques on average than those graduating in other years (P<0.05). Similar to family physicians, pediatricians that had taken a communications course outside of medical school used significantly more of the 17 total and seven basic communications techniques on average than those who had not (17 techniques: 6.96 vs. 5.84, P≤0.01; seven basic techniques: 3.67 vs. 2.83, P≤0.001). Finally, a significantly greater mean number of techniques were observed in pediatricians that had assessed their office for user-friendliness than those who had not (P≤0.001). All pediatrician practice characteristics showed a significant association with the mean number of both the 17 and the seven basic techniques used. A significantly higher mean score was observed as the percentage of pediatricians’ child patients on Medicaid increased (P <0.05). Additionally, pediatricians who practiced in settings other than group or solo practices (i.e.: public health, hospital, other) and those who had an occupation other than a private practitioner reported significantly greater mean use of the 17 total and the seven basic communication techniques.

Tables 7 and 8 present results from the ordinary least squares regression analysis with communication techniques as the dependent variable. The observed results were generally in-line with the associations observed in the bivariate regression analysis, with some additional information provided. As seen in the bivariate analysis, family physicians who had taken a communications course were more likely to use the 17 techniques than those who did not (P<0.01). Family physicians with less than 26% of their child patients on Medicaid were significantly less likely to use the 17 techniques and the seven basic techniques compared to those providers who had greater than 75% of their patient population on Medicaid (P<0.05).

**Table 7:** Ordinary least squares regression of predictor variables on number of communication techniques used by family physicians.

Variable	17 communication techniques (n = 79)		Seven basic communication techniques (n = 79)	
	Coefficient (Standard Error)	P-value	Coefficient (Standard Error)	P-value
**Year of Graduation**				
1979 or prior	-1.84 (1.48)	0.22	-0.28 (0.74)	0.70
1980–1989	-0.92 (1.14)	0.42	-0.31 (0.56)	0.59
1990–1999	-1.82 (1.20)	0.14	-0.42 (0.60)	0.49
2000 or later (Ref)	1.00	N/A	1.00	N/A
**Race/Ethnicity**				
Black	0.10 (1.70)	0.95	-0.60 (0.85)	0.48
All Other	2.14 (0.99)	0.03*	0.15 (0.49)	0.77
White (Ref)	1.00	N/A	1.00	N/A
**Gender**				
Female	0.83 (0.82)	0.32	-0.003 (0.40)	0.99
Male (Ref)	1.00	N/A	1.00	N/A
**Ever taken a communications course**				
Yes	2.21 (0.75)	0.004**	0.53 (0.38)	0.16
No (Ref)	1.00	N/A	1.00	N/A
**Assessed office for user friendliness**				
Yes	0.64 (0.80)	0.43	-0.010 (0.39)	0.98
No (Ref)	1.00	N/A	1.00	N/A
**Percentage of pediatric patients with Medicaid**				
0 to 25%	-2.31 (1.11)	0.04*	-1.20 (0.56)	0.04*
26 to 50%	-0.98 (1.43)	0.50	-0.64 (0.72)	0.37
51% to 75%	-1.00 (1.70)	0.56	-0.20 (0.86)	0.82
76% to 100% (Ref)	1.00	N/A	1.00	N/A
**Practice setting**				
Group practice	0.31 (1.12)	0.78	-0.36 (0.54)	0.51
All other	0.96 (1.18)	0.42	-0.49 (0.57)	0.39
Solo practice (Ref)	1.00	N/A	1.00	N/A
**Primary occupation**				
All other	0.63 (0.83)	0.45	-0.34 (0.40)	0.39
Private practice (Ref)	1.00	N/A	1.00	N/A

*P <0.05

**P≤0.01

**Table 8:** Ordinary least squares regression of predictor variables on number of communication techniques used by pediatricians.

Variable	17 communication techniques (n = 215)		Seven basic communication techniques (n = 215)	
	Coefficient (Standard Error)	P-value	Coefficient (Standard Error)	P-value
**Year of Graduation**				
1979 or prior	0.23 (0.64)	0.72	0.24 (0.31)	0.44
1980–1989	-1.00 (0.66)	0.13	-0.50 (0.32)	0.12
1990–1999	-1.30 (0.68)	0.06	-0.49 (0.33)	0.14
2000 or later (Ref)	1.00	N/A	1.00	N/A
**Race/Ethnicity**				
Black	1.34 (0.77)	0.09	0.45 (0.38)	0.25
All Other	-0.18 (0.62)	0.77	0.22 (0.31)	0.48
White (Ref)	1.00	N/A	1.00	N/A
**Gender**				
Female	0.25 (0.45)	0.57	-0.002 (0.22)	0.99
Male (Ref)	1.00	N/A	1.00	N/A
**Ever taken a communications course**				
Yes	1.11 (0.43)	0.01**	0.84 (0.21)	<.0001***
No (Ref)	1.00	N/A	1.00	N/A
**Assessed office for user friendliness**				
Yes	1.43 (0.43)	0.001***	0.74 (0.21)	0.001***
No (Ref)	1.00	N/A	1.00	N/A
**Percentage of pediatric patients with Medicaid**				
0 to 25%	-1.77 (0.62)	0.01**	-0.98 (0.30)	0.001***
26 to 50%	-1.39 (0.73)	0.06	-0.87 (0.35)	0.01*
51% to 75%	-1.23 (0.84)	0.15	-0.70 (0.41)	0.09
76% to 100% (Ref)	1.00	N/A	1.00	N/A
**Practice setting**				
Group practice	1.01 (0.67)	0.13	0.33 (0.34)	0.33
All other	2.56 (0.74)	0.001***	0.98 (0.37)	0.01**
Solo practice (Ref)	1.00	N/A	1.00	N/A
**Primary occupation**				
All other	1.48 (0.47)	0.002**	0.76 (0.23)	0.001***
Private practice (Ref)	1.00	N/A	1.00	N/A

**P≤0.01

***P≤0.001

In agreement with what was observed in the bivariate analysis, pediatricians who had taken a communications course or assessed their office for user-friendliness were more likely to use the 17 techniques and the seven basic techniques than those providers who had not. Likewise, pediatricians with less than 26% of their child patients on Medicaid were less likely to use the 17 techniques and seven basic techniques than those who had greater than 75% of their patient population on Medicaid (P≤0.01 and P≤0.001, respectively). Pediatricians in practice settings other than solo or group practices were more likely to use the recommended communications techniques than their counterparts, as were pediatricians who had an occupation other than a private practitioner.

## Discussion

### Routine use of communication techniques

The AMA and health literacy experts have recommended 17 techniques that physicians can use to improve communication with their patients [11]. Similar to previous studies, this study found that many of these communication techniques were under-utilized by Maryland physicians [11,15,16]. Family physicians and pediatricians make up a large majority of primary care providers in the U.S., and although slight differences were observed, overall the two groups of physicians in this study were similar in their low, routine use of many of the recommended communication techniques.

The use of simple language was the technique that both groups of physicians in this study used most routinely and felt was most effective. Using simple language and avoiding jargon has been previously demonstrated to have a high utility amongst physicians [11]. While it is encouraging to see that many physicians focus on using simple language, previous research has also found that physicians tend to underestimate their use of jargon during patient encounters even if unknowingly [16]. This study relied on self-report on use rather than direct observation, so there was no way to objectively determine how ‘effectively’ these techniques were delivered. Future studies should consider not only evaluating the use of these techniques, but also whether these communication techniques were used effectively.

A concerning finding was that only approximately 30% of both family physicians and pediatricians in this study routinely used the ‘teach-back methods’ of asking patients to repeat back information and asking patients to tell them what they will do at home to follow instructions. This finding is important because ‘teach-back methods’, which assess patients’ understanding of information received by having them repeat it, are strategies that the Institute for Healthcare Improvement and health literacy experts recommend all healthcare providers use [22]. The low routine use comes in spite of the fact that over 60% of physicians in the sample felt that these teach-back techniques were effective. Additionally, many physicians were unsure of the effectiveness of several of the recommended techniques, which could suggest that physicians are unaware of valuable skills that could enhance their communication. Taken together, these findings suggest that provider communication skills should receive a higher priority in the medical and residency training process, including continuing medical education course offerings; so that physicians have the opportunity to more regularly utilize and refine these skills over the course of their careers, thereby making them more likely to use them in practice settings.

### Factors affecting use of communication techniques

Both pediatricians and family physicians in this study who had taken a communications course used more communications techniques than their counterparts. This finding provides additional support for increasing physicians’ exposure to communications training as a way to increase their utilization of communication techniques.

The physicians with a high percentage of patients on Medicaid were also more likely to use the recommended communications techniques (total and seven basic) than those physicians with a low percentage of patients on Medicaid. Additionally, pediatricians who served in non-private practice settings (i.e. public health centers, hospitals) were more likely to use recommended communication techniques than their counterparts. This is a positive finding given that health literacy has been observed to be lower amongst underserved individuals [3]. Physicians may naturally place a greater emphasis on communicating effectively with underserved populations, however, it is important for physicians to realize how pervasive low health literacy is for many different segments of the population [3]. If physicians were more aware of the preponderance of low health literacy in the population and its effects on patient outcomes, they may be more motivated to utilize effective communication techniques for all patients as a regular part of their practice. As part of communications training, physicians should also be made aware of the prevalence of low health literacy in the population and its negative effects on health outcomes [5–9].

### Study limitations

There are important limitations in this study. Although the 20% response rate in this study is typical of many mailed surveys to healthcare providers [23], it likely introduced some selection bias into the study, where participating physicians’ responses may not reflect the views of non-responders. Providers who participated in this study were likely to be more interested in the study topic than those who did not, and information on the characteristics of non-responders could not be obtained. Another limitation of this study was the use of a self-reported questionnaire to determine physicians’ communication practices as opposed to using direct observation to validate communication. Although physicians may over-estimate their use of recommended communications techniques, using a validated survey allowed for greater study efficiency and the analysis of a larger sample size. Despite the limitations, this study provides strong baseline data that can be used to develop and implement educational interventions and policies in Maryland aimed at enhancing provider communication.

## Conclusions

The purpose of this study was to determine the use of recommended communication techniques by family physicians and pediatricians in Maryland. Overall, and in agreement with previous studies, the use of communication techniques by physicians in the study sample was low [11,15,16], however, those physicians with additional communications training (i.e. those who had taken a communications course) used more techniques than those who had not. The benefits of effective communication have been well documented [12–14]. Additionally, because of the wide range of patients that family physicians and pediatricians see, these providers have the opportunity to communicate and promote other aspects of their patients’ health such as oral health, making their effective communication especially important [18,19].

One way to help achieve the Healthy People 2020 objective of increasing satisfactory communication by providers, would be to increase the priority of communications training in the medical education process [2]. Potential ways to incorporate more communications training could include: having trained faculty provide more observation and feedback of medical students’ communication with patients during their clinical years (years 3 and 4); having a greater focus on the Accreditation Council for Graduate Medical Education (ACGME)-required interpersonal and communication competency during residency training through the use of patient and faculty feedback; and incorporating required continuing medical education in communication as part of the maintenance of certification [2]. As the focus on patient-centered care in the U.S. increases, the need for effective provider communication will become even more essential. Incorporating this skill into the life-long learning process of physicians and other health care providers will help to ensure that they are properly trained in this important aspect of patient care.

## Supporting Information

S1 Document Physician Survey. This is a modified questionnaire that was used to survey the Family physicians and Pediatricians in this study on their communication practices. This survey also captures Provider and Practice demographic information. Because this survey was used for a larger study that included dental caries prevention, only the questions pertaining to provider communication are included (the questions related to dental caries prevention have been removed). Each question corresponds to a variable in the databases. Both Family physicians and Pediatricians used the exact same questionnaire in this study.
(DOCX)

S1 Dataset Family Physicians Dataset. This is the dataset that contains the data collected from Family physicians using the Physician Survey. The variables in the dataset begin with a Q followed by a number, which corresponds to the specific question in the Physician survey. Some questions contain sub-questions, and these corresponding variables contain an underscore after the question number followed by a number/letter to denote the sub-question. Furthermore, for Question 12 on the Physician Survey, participants answered a) How often they use communication techniques, and b) Do they think it is effective, which are denoted by an “a” or “b” in the variable. The values of the variables in the dataset correspond to the numbers listed next to the answer choices on the Physician Survey.
(XLS)

S2 Dataset Pediatricians Dataset. This is the dataset that contains the data collected from Pediatrician using the Physician Survey. The variables in the dataset begin with a Q followed by a number, which corresponds to the specific question in the Physician Survey. Some questions contain sub-questions, and these corresponding variables contain an underscore after the question number followed by a number/letter to denote the sub-question. Furthermore, for Question 12 on the Physician Survey, participants answered a) How often they use communication techniques, and b) Do they think it is effective, which are denoted by an “a” or “b” in the variable. The values of the variables in the dataset correspond to the numbers listed next to the answer choices on the Physician Survey.
(XLS)
