# Supplementary figures and images for: The Use of Recommended Communication Techniques by Maryland Family Physicians and Pediatricians
Source: PLoS One. 2015 Apr 9;10(4):e0119855. doi: 10.1371/journal.pone.0119855 (PMC4391842; doi:10.1371/journal.pone.0119855)

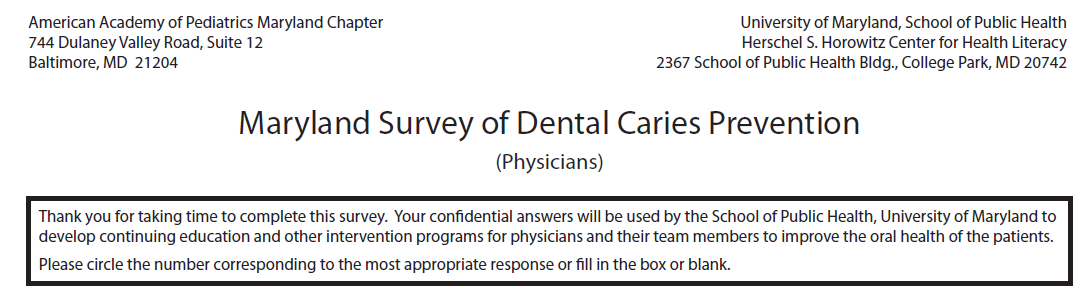


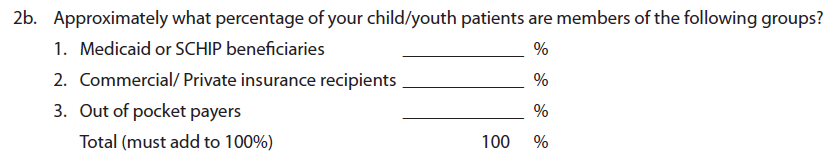


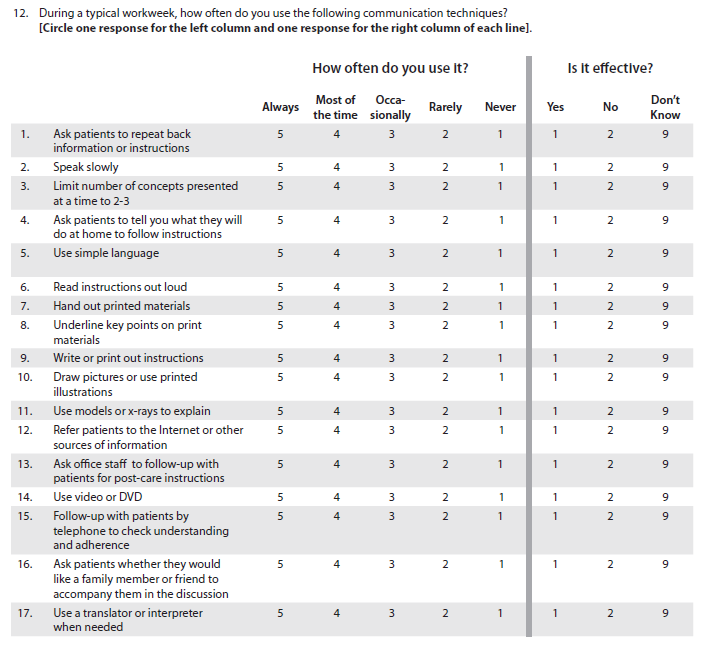


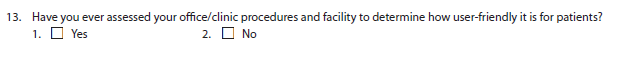


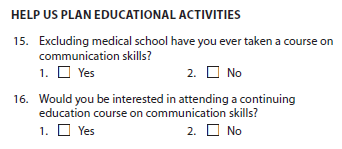


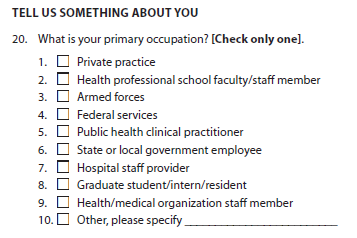


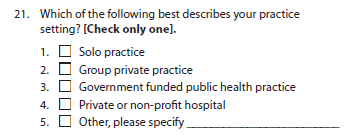


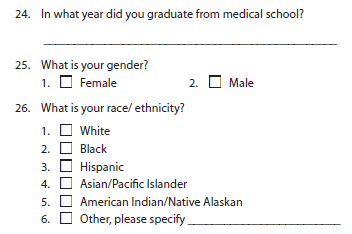

Supplement: S1 Document — This is a modified questionnaire that was used to survey the Family physicians and Pediatricians in this study on their communication practices. This survey also captures Provider and Practice demographic information. Because this survey was used for a larger study that included dental caries prevention, only the questions pertaining to provider communication are included (the questions related to dental caries prevention have been removed). Each question corresponds to a variable in the databases. Both Family physicians and Pediatricians used the exact same questionnaire in this study. (DOCX) [file pone.0119855.s001.docx]
